# Re-Audit of Discontinuation Plans on Discharge for Prescribed Hypnotics in General Adult Inpatient Services

**DOI:** 10.1192/bjo.2025.10659

**Published:** 2025-06-20

**Authors:** Rifat Binte Radwan, Aamir Mujtaba

**Affiliations:** Essex Partnership University NHS Foundation Trust, Essex, United Kingdom

## Abstract

**Aims:** Assess the number of patients discharged from General Adult Psychiatry wards with hypnotics prescribed for insomnia.

Evaluate the consistency of documenting discontinuation plans for hypnotics in discharge summaries.

**Methods:** We conducted a retrospective review of 24-hour discharge summaries for patients discharged from six adult inpatient general psychiatry wards between 15/01/2024 and 15/04/2024. The review focused on patients prescribed regular hypnotics for insomnia, specifically analysing the “Instructions to GP” section to determine whether a specific discontinuation plan was recommended or advised. This was done by reviewing our online database used within the trust.

**Results:** In the current audit, 56.25% of the sample had discharge summaries that included a medication review for hypnotics suggested to the GP, while only 18.75% included a specific discontinuation plan for hypnotic medication. A previous audit conducted in 2023 on two adult inpatient general psychiatry wards demonstrated 0% compliance, with no discharge summaries containing a medication review for hypnotics or a specific discontinuation plan. Following the implementation of changes, a re-audit in 2024 on the same wards showed significant improvement, with 66.6% of discharge summaries including a medication review for hypnotics and 33.3% containing a specific discontinuation plan for hypnotic medication.

**Conclusion:** The previous recommendations have led to noticeable improvements; however, strict adherence to these recommendations is necessary to achieve the target of 100% compliance. It is crucial for the inpatient General Adult Psychiatry team to consistently communicate a specific discontinuation plan for hypnotics to the GP. This practice is essential to reduce the risk of dependence and minimize potential side effects associated with hypnotic medications.

